# Embracing the Heterogeneity Within Particulate and Mineral‐Associated Organic Matter for the Next Generation of Soil Carbon Questions

**DOI:** 10.1111/gcb.71191

**Published:** 2026-11-04

**Authors:** Sam J. Leuthold, Jocelyn M. Lavallee, M. Francesca Cotrufo

**Affiliations:** ^1^ Soil Innovation Laboratory, Department of Soil and Crop Sciences Colorado State University Fort Collins Colorado USA; ^2^ Environmental Defense Fund New York New York USA

## Abstract

Soil organic matter (SOM) is a complex and dynamic substrate central to the functioning of terrestrial ecosystems. Because of its heterogeneity, SOM is routinely parsed into physical fractions with more uniform composition and behavior. Over the last several years, particulate (POM) and mineral‐associated (MAOM) organic matter have become the most widely adopted of these fractions, which has given the field a shared, comparable framework for resolving the first‐order heterogeneity within SOM. As fractionation has become routine, however, it has become clear that POM and MAOM identity alone cannot resolve the finer, second‐order variation that additional mechanistic inference requires. This is in part because the fractions are operationally defined composites, whose properties vary across composition, formation pathway, and behavior. Conceptualizing them as the terminal state of analysis collapses each fraction to its central tendency, flattening the distribution and obscuring the structure on which more complex questions depend. Here we propose embracing this within‐fraction heterogeneity rather than averaging over it, treating POM and MAOM as composite distributions whose internal structure underlies the variation in how they form, persist, and function. We suggest that this view can be operationalized via two complementary approaches: tailoring fractionation methods to target subpopulations most relevant to a question, and pairing fractions with orthogonal measurements. Together, these resolve the within‐fraction distribution that bulk measurements integrate over, narrowing the analysis onto the most explanatory slice of the bulk SOM. Similar to the original framework, we do not aim to prescribe a certain methodology, but rather a way of thinking about heterogeneity within SOM, in which a separation is justified by the understanding it produces rather than the data it generates. This way of reasoning allows POM and MAOM to continue linking organic matter to its functions, and in doing so supports more informed stewardship of soil carbon.

## Graphical abstract

Measurements of particulate (POM) and mineral‐associated organic matter (MAOM) alone can collapse within‐fraction variability to a central tendency. Embracing this internal heterogeneity enables more complex questions, in which POM and MAOM are viewed as composite distributions characterized through refined fractionation or orthogonal measurements, better connecting fractions to the next set of questions.

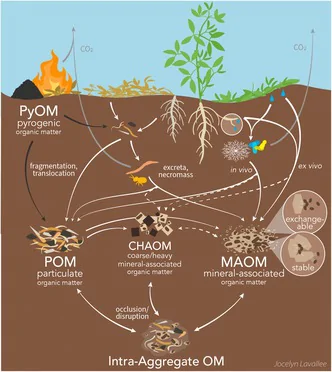

## 1 Introduction

Soil organic matter (SOM) underpins biogeochemical functioning in terrestrial ecosystems, regulating nutrient cycling, water retention, and carbon (C) storage. SOM has been studied for more than a century (Waksman 1936), yet its internal heterogeneity has made it difficult to characterize as a single, coherent material (Schmidt et al. 2011; von Lützow et al. 2007). Physical fractionation is one method that attempts to parse this variability and has been extensively employed to infer how SOM forms, what it consists of, how it persists, and what drives its turnover. While the earliest applications focused on separating materials of different composition and origin (primarily as preparation for compositional analyses such as humic extraction; Greenland and Ford 1964; Ford et al. 1969), fractionation has since expanded into one of the core methods for resolving SOM heterogeneity (Cambardella and Elliott 1992; Christensen 2001; Cotrufo and Lavallee 2022). With that expansion came a proliferation of fractionation methods, developed largely in parallel to each other in response to the range of mechanistic questions fractionation could answer (Poeplau et al. 2018). The diversity of approaches advanced understanding of key soil biogeochemical processes, including aggregate occlusion (Jastrow 1996; Six et al. 2004), mineral sorption (Sollins et al. 1984; Kleber et al. 2007), and SOM formation (Cotrufo et al. 2015). However, the diversity also raised the barrier to entry, as new methods were time intensive, isolated various fractions that were not designed to be interchangeable and created a significant semantic ambiguity as different soil fractions often shared the same name and similar conceptual definitions. As the humic acid paradigm for understanding SOM receded (Lehmann and Kleber 2015; Baveye and Wander 2019), the field needed to converge around a shared foundational structure for understanding SOM complexity (Rocci et al. 2024).

Lavallee et al. (2020) codified what the community was converging around, recommending particulate organic matter (POM) and mineral‐associated organic matter (MAOM) as the primary fractions of inquiry. This gave the community a shared foundation and allowed researchers in related fields to engage with SOM heterogeneity without substantial investment in new equipment. The shared language of POM and MAOM enabled synthesis at new scales, from continental to global (Sokol et al. 2022; Hansen et al. 2024; Yu et al. 2022), partly because more researchers adopted these relatively simple fractionation schemes, increasing the amount of fraction data available. It also provided targets for a new set of process‐based models, with pools that could be measured directly rather than estimated (Robertson et al. 2019; Zhang et al. 2021; Abramoff et al. 2018). The framework was widely adopted largely because it captured the core heterogeneity within SOM while remaining simpler than many preceding fractionation approaches (Rocci et al. 2024).

The dichotomy of POM and MAOM was designed to resolve first‐order heterogeneity in SOM, separating it into two fractions that reflect major contrasts in formation pathway, composition, and behavior. Lavallee et al. (2020) chose this resolution deliberately, prioritizing parsimony and a harmonizing structure over greater within‐fraction detail, while noting that specialized fractionations were still necessary for some questions. That parsimony is the POM‐MAOM framework's strength, but it also defines the outer bound of its domain of validity. Where questions require categorizing SOM by broad formation pathways (structural vs. soluble inputs; Cotrufo et al. 2015), capacity of accrual (i.e., effectively unbounded vs. finite; Six et al. 2002; Cotrufo et al. 2019; King et al. 2024), or temperature sensitivity (depolymerization vs. sorption–desorption; Conant et al. 2011; Lugato et al. 2021; Georgiou et al. 2024; Sun et al. 2026), the framework remains a firm basis for inference. Where interpretation depends on within‐fraction variation along axes such as composition (Angst et al. 2021; Yu et al. 2022), persistence (Stoner et al. 2023; Jilling et al. 2025), vulnerability (Jilling et al. 2020; Díaz‐Martínez et al. 2024), or relationship to ecosystem function (Ros et al. 2011; Ros 2012; Curtin et al. 2019), the dichotomy fails to resolve the distributions that matter most. In these cases, the commonly isolated fractions may fail the homogeneity and uniqueness criteria of a robust conceptual pool, limiting the ability to draw conclusions from them (Curtin et al. 2019).

Here we take stock of the POM‐MAOM framework in its current form and define its domain of validity. We then expand the framework by conceptualizing POM and MAOM as composite distributions: collections of SOM subpopulations which vary in composition, persistence, function, and vulnerability to change, but are isolated together during physical fractionation. Any property measured on the whole fraction therefore represents a central tendency across the various subpopulations. Finally, we propose how this view can be operationalized by narrowing and characterizing those distributions to address the next set of questions about SOM.

## 2 Physical Fractionation's Domain of Validity

POM and MAOM are not measured directly; they are approximated from operational proxies. Conceptually, the two fractions represent distinct pools of SOM, expected to differ in formation pathways, dominant constituents, and average behavior. In practice, physical fractionation operationalizes these pools using particle size and/or density, two properties that broadly covary with the degree and mode of association between organic matter and mineral surfaces (Poeplau et al. 2018; Leuthold et al. 2023, 2024). The fractions it produces are mixtures of mineral and organic material, not organic matter alone. Material denser than 1.6–1.8 g cm^−3^ or smaller than 20–53 μm is generally classified as MAOM, and the coarser or less dense residual as POM (Lavallee et al. 2020). The thresholds sort whole particles by their ability to form mineral associations, assigning each a fraction identity without measuring the defining association directly. Reading composition, persistence, function, or vulnerability from these categories rests on the further assumption that those same properties differ between the fractions isolated by this criterion (Cambardella and Elliott 1992; Christensen 2001). The framework is strongest where that assumption holds. However, it begins to break down when fraction identity is used as a stand‐in for properties that the separation does not directly resolve, such as when an increase in MAOM C is perceived as an increase in “permanent” soil C.

The thresholds have largely succeeded in isolating distinct fractions. Yet because they rely on physical proxies, different fractionation methods produce groupings that are similar but not identical (Kpemoua et al. 2026; Leuthold et al. 2024; Just et al. 2021). Even within the same method, thresholds can interact with soil properties, muddling comparisons among fractions that share the same name and methodological provenance (Motsi et al. 2026; Poeplau et al. 2025). Recovery, too, is method‐ and soil‐dependent. Reported recoveries of organic C often fall well below 100%, reflecting soluble losses, transfer artifacts, or material that does not partition cleanly into either fraction—variability that is rarely accounted for when fraction stocks are compared across studies. These tensions extend further in some soils, breaking the binary pool assumption entirely. In Andisols, for example, pseudo‐sand structures of short‐range‐order minerals and Al‐OM complexes place mineral‐associated material in size classes the framework assigns to POM (Kilian Salas et al. 2024); in highly structured soils, stable aggregates obscure whether material is mineral‐bound or simply occluded (Breure et al. 2026); and in highly sandy soils, low reactive mineral surface area means the binary is poorly suited to resolve differences (Poeplau et al. 2025). In each case here, the binary POM–MAOM conceptualization fails as an organizing structure.

These examples point to the central tension of operational fractions: there is no procedure‐independent POM or MAOM against which methods can be calibrated. If the fractions were fixed natural entities, then differences in density threshold, dispersion intensity, soil mineralogy, or recovery efficiency would have limited influence on what was isolated. Instead, these choices, along with fixed edaphic properties, partly determine the material assigned to each fraction. Our procedures therefore produce operational approximations of POM and MAOM rather than discovering invariant entities. This does not make the fractions arbitrary; it means their inferential value depends on whether the approximation is adequate for the question being asked.

Nevertheless, meaningful, mechanistically grounded differences in the average chemical composition and behavior of the fractions remain a useful tool. On average, for instance, POM has a higher C:N ratio and is derived from structural polymeric compounds, while MAOM tends to be composed of low‐molecular weight compounds with longer mean residence times (Cotrufo and Lavallee 2022). The challenge arises when those empirical tendencies are solidified into fixed identities that the separation cannot guarantee. The properties attributed to each fraction are central tendencies of distributions, not fixed rules. What the POM–MAOM framework can answer then depends less on a question's topic than on the resolution it demands. Questions about a fraction's average behavior, such as the general drivers of formation (Hansen et al. 2024; Viscarra Rossel et al. 2019), mean turnover time (Heckman et al. 2022), or bulk stoichiometry (Christensen 2001; Cotrufo et al. 2019), are captured by the central tendency, and here the framework is a firm foundation. However, some questions implicitly target a specific subpopulation: which forms of C persist, or which drive a particular ecosystem function, for example. These require resolving the within‐fraction distribution that the bulk measurements integrate over, where the central tendency, while still informative, is no longer fully sufficient.

## 3 The Distributional Structure of POM and MAOM

POM and MAOM are heterogeneous composites, not internally uniform pools. Within each fraction, properties vary along different dominant axes: POM with the source and chemistry of its inputs, MAOM with the nature of its organo‐mineral associations (bonding configuration, mineral assemblage, microbial accessibility), and both with physical occlusion and the exchange it mediates between them (Fulton‐Smith et al. 2024). Because the pools correspond to these axes rather than determine them, fraction‐level properties form distributions along the axis of variation (Figure 1). A bulk measurement flattens that distribution into a central tendency, while characterizing its shape can reveal the structure the average obscures (Figure 1).

**FIGURE 1:**
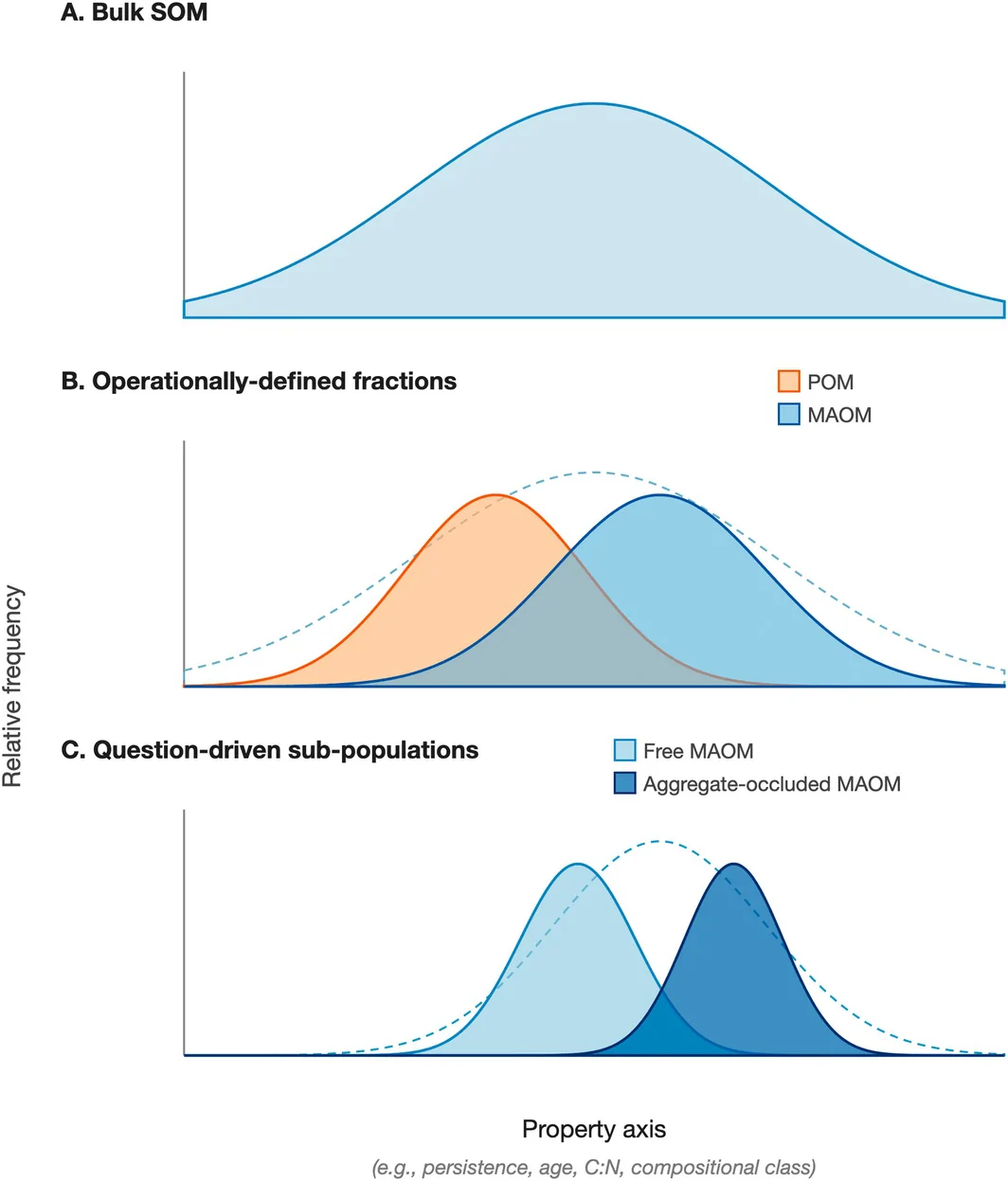
Within‐group heterogeneity is broadest at the bulk soil organic matter (SOM) level, narrows partially with fractionation, and narrows further as further analyses resolve the distribution along the axis a question concerns. Distributions are conceptualized as relative frequency along a generic property axis, such as persistence, radiocarbon age, C:N, or compositional class. (A) Bulk SOM spans a single broad distribution, a wide internal spread of property values summarized by one central tendency. (B) Operational fractionation splits it into POM and MAOM, two narrower, partially overlapping composite distributions whose central tendencies differ even as their ranges overlap. (C) Subfractionation resolves a fraction into question‐relevant subpopulations (e.g., free and aggregate‐occluded MAOM), each narrower than the parent fraction.

### 3.1 Origins of Within‐Fraction Variation

The distributed nature of SOM fractions emerges from the diversity of formation pathways, biological inputs, and binding mechanisms collapsed into each operational fraction. Plant, microbial, and pyrogenic inputs all enter both POM and MAOM through a range of formation pathways (Cotrufo et al. 2015; Sokol et al. 2019; Cotrufo et al. 2024), with fire‐altered material contributing a distinct, aromatic compositional class to each (Figure 2; Bird et al. 2015; Leuthold, Soong, et al. 2025). At the first‐order scale, the inputs routed to each fraction are relatively distinct. POM forms largely from structural, polymeric material, while MAOM forms from low‐molecular weight, soluble compounds, either directly, or after microbial assimilation. Yet, each of these pathways carries substantial internal variation that the fraction‐level distinction integrates over. Aboveground litter turnover and the post‐mortem decay of root tissue both undergo fragmentation and contribute plant material to POM (Cotrufo and Lavallee 2022; Kätterer et al. 2011), while fungal necromass and cell wall residues add microbial‐derived inputs to the POM fraction (Witzgall et al. 2021; Klink et al. 2022). MAOM formation is similarly heterogeneous: along the in vivo pathway, organic matter is routed through microbial anabolism, with the resulting necromass and extracellular byproducts deposited onto mineral surfaces (i.e., the microbial carbon pump; Liang et al. 2017, 2019). The ex vivo formation pathway, by contrast, delivers low‐molecular weight, soluble compounds directly to mineral surfaces, bypassing microbial assimilation (Figure 2; Sokol et al. 2019; Cotrufo et al. 2015, 2024). Beyond the pathway distinction, the binding mechanisms themselves vary, including surface complexation, ligand exchange, cation bridging, and the zonal multilayer accumulation of organic compounds on mineral surfaces (Kleber et al. 2007, 2021; Mikutta et al. 2006). Similarly, the mineral phase varies between soils, with important consequences for organo‐mineral bonding. Iron and aluminum oxides, phyllosilicate clays, and poorly crystalline minerals differ in surface area, charge, and reactivity, producing different expressions of these binding mechanisms and different saturation states depending on the mineral assemblage (Rasmussen et al. 2018; Kleber et al. 2015; Georgiou et al. 2022). Across both POM and MAOM, formation pathway is an important axis of within‐fraction variability.

**FIGURE 2:**
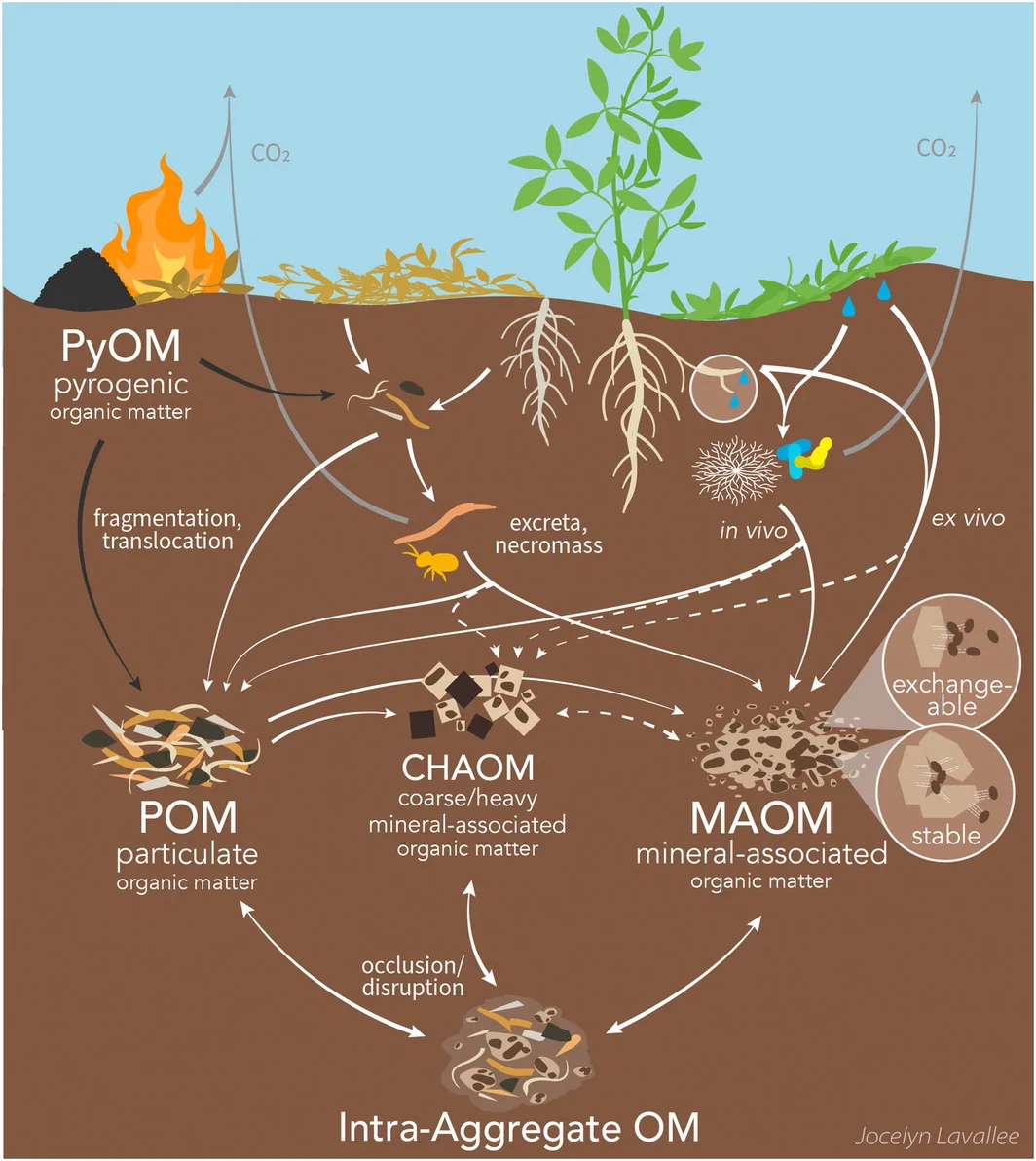
Soil organic matter resolved beyond the POM/MAOM dichotomy. Structural compounds of plant, microbial, or pyrogenic (PyOM) origin form POM, while MAOM originates from low‐molecular weight, water‐soluble compounds through a variety of processes. Above and belowground litter fragments into particulate organic matter (POM); microbial excreta and necromass build mineral‐associated organic matter (MAOM) in vivo, while simultaneously structural microbial components are incorporated into POM. Dissolved soluble organic matter sorbs to mineral directly ex vivo. These multiple pathways cut across fractions, such that the organic matter associated with coarse, heavy minerals (CHAOM) occupies a distinct position between POM and MAOM rather than collapsing into either, and PyOM distributes across all three. Physical occlusion within aggregates couples the fractions via occlusion, and MAOM itself spans a continuum from exchangeable to stable forms. These cross‐fraction interactions reveal the second‐order heterogeneity key to understanding SOM function. Figure by Jocelyn Lavallee.

Partly as a consequence of the variability in formation pathways, POM and MAOM contain significant internal compositional heterogeneity, despite the common characterization of POM as more plant‐derived and MAOM as more microbial‐derived. This heuristic reflects an over‐reliance on the central tendency of either fraction and does not extend to individual ecosystems or specific soils. Indeed, the underlying distributions of the internal composition of POM and MAOM are often broad and partially overlapping, with the balance between plant‐ and microbial‐derived material within a fraction shifting across edaphic contexts (Figure 1; Whalen et al. 2022; Yu et al. 2022). Compositional heterogeneity is further compounded in a dichotomous framework by operational definitions, which group compositionally distinct material into a single class, particularly under one‐step size‐ or density‐based fractionation (Leuthold et al. 2024). Organic matter separated with the coarse heavy (> 53 μm and > 1.85 g cm^−3^) soil fraction (CHAOM), for example, is commonly grouped with size‐separated POM despite its association with coarse, dense mineral material and its distinct compositional signature (Figure 2; Samson et al. 2020). Likewise, pyrogenic organic matter (PyOM) distributes across both POM and MAOM rather than sorting cleanly into one (Figure 2), its cyclic, relatively persistent chemistry shifting each fraction's distribution toward higher aromaticity and longer apparent turnover times (Bird and Ascough 2012; Lavallee et al. 2019; Leuthold, Soong, et al. 2025). Within‐fraction variation is also structured by the distribution of binding strength and by the physical accessibility of organic matter to decomposition (Figure 2). Though often recognized (e.g., Jilling et al. 2025), this variability has not yet been widely formalized in how fractions are interpreted or measured. Compositional variation within a fraction is often left unresolved, and chemically distinct materials tend to be grouped together by whatever procedure isolated them. Rather than treating a given fraction property as uniform, a distributional view inherently recognizes the natural variation within composite fractions and uses it as an explanatory axis (Figure 1).

### 3.2 Stability, Vulnerability, and Function of SOM Fractions

Persistence is another axis along which within‐fraction variation shapes inference. Although fraction identity provides a broad ordering, residence times within either POM or MAOM can span orders of magnitude, varying with mineral assemblage and physical occlusion (Heckman et al. 2018; Torn et al. 1997). In MAOM, exchangeable forms can cycle on seasonal timescales (Jilling et al. 2025), while strongly bound, mineral‐stabilized material can persist for centuries (Kleber et al. 2021). Persistence in POM is similarly variable; physical occlusion within aggregate microsites can extend POM turnover from years to decades, particularly under oxygen‐limited conditions (Keiluweit et al. 2017), or in energy limited, low‐temperature regions (e.g., permafrost; García‐Palacios et al. 2024). Vulnerability under disturbance varies along the same axis. Warming, tillage, or shifting moisture regimes mobilize free POM and exchangeable MAOM most rapidly, while occluded POM and strongly mineral‐bound MAOM are more protected (Lugato et al. 2021; Kleber et al. 2021). The persistence and vulnerability of these fractions tend to vary along a continuous axis within each, shaped by the mechanisms of stabilization and destabilization. Along that axis, POM's median sits nearer the vulnerable end and MAOM's nearer the persistent end, even as the two distributions overlap (Figure 1). Answering questions about persistence or vulnerability thus depends as much on where a subpopulation sits along that axis as on which fraction it occupies.

Questions about ecosystem function, such as crop yield, nutrient provisioning, or water retention, shift the focus from the properties of a fraction to its consequences. Here, within‐fraction variation often determines what fraction‐level inference can support. Because a single fraction can comprise material of widely varying origin, chemistry, and susceptibility to decomposition, bulk process rates become weighted averages of very different subpopulations. At this level of inference, fraction identity weakly predicts nitrogen (N) mineralization rates (Ros et al. 2011; Ros 2012). The behaviors expected from the central tendency can even reverse: more N can be mineralized from MAOM than from POM (Sollins et al. 1984; Villarino et al. 2023; Leuthold, Lavallee, et al. 2025), and POM can decompose more slowly than MAOM (Crow et al. 2007). The relationship between fraction abundance and outcomes such as crop yield has proven inconsistent, with MAOM positively associated with yield in some systems and negatively in others (Wood et al. 2016, 2018), and POM showing no significant relationship at all (Cates and Ruark 2017). We posit that these inconsistencies arise because what drives function lies in the within‐fraction structure, not in the fraction‐level average. Function may therefore be the axis along which fraction identity is least diagnostic.

### 3.3 From Fraction Identity to Distributional Inference

The original framework resolves the first‐order heterogeneity in SOM (i.e., POM and MAOM). Combining fraction identity with the shape of the within‐fraction distribution extends that resolution into the second order (e.g., free vs. occluded POM and MAOM; Figure 1). The combination preserves the shared language of POM and MAOM while opening the door to deeper questions about what SOM is and what it does. Two distinct questions follow. One concerns the shape of the within‐fraction distribution: how broadly and how evenly do properties vary across the fraction? The other concerns position: which part of that distribution does a given subpopulation occupy? The first is answered by characterizing the distribution; the second, by narrowing it to the relevant subpopulation. This is the basis of an inquiry‐driven approach to fraction studies, in which the question determines which axis of variation matters most. Decades of fractionation research, more recently codified by the POM‐MAOM framework and expanded by subsequent syntheses, have built the conceptual vocabulary that now makes these targeted, within‐fraction hypotheses possible.

## 4 Towards an Inquiry‐Driven Framework

Analyses of SOM often stop at POM and MAOM, effectively treating the fractions as a terminal state of resolution. Consequently, inference is most often fixed at the fraction level, leaving the within‐fraction structure and second‐order heterogeneity unresolved. A distributional view treats POM and MAOM as starting points instead of endpoints. It acknowledges the within‐fraction variability and pursues two complementary objectives: narrowing the distribution onto question‐relevant subpopulations and characterizing it along the axes most relevant to the question (Figure 1). Such inquiry requires going beyond POM‐MAOM separation by further refining and expanding physical fractionation procedures, or by pairing fractions with orthogonal measurements, which characterize properties along the relevant axis that the dichotomous separation cannot resolve (Figure 3). This approach aims to shift the balance of our measurements from differences between fractions toward variation within them, with the goal of connecting that variation to theories of biogeochemical function (Baveye 2025).

**FIGURE 3:**
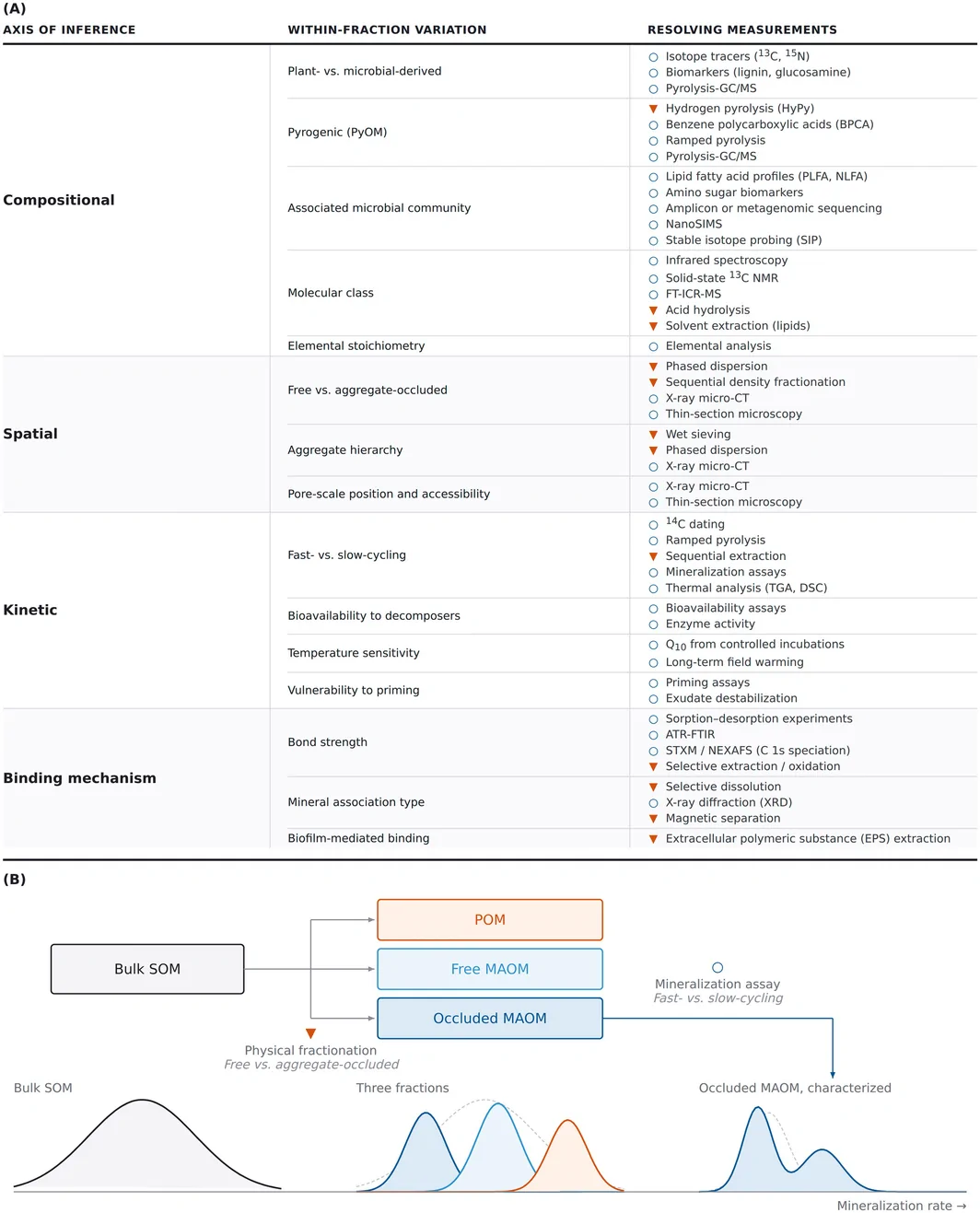
Methods available for resolving within‐fraction variation, and their application to an example question. (A) Within‐fraction variation grouped by axis of inference, each paired with measurements capable of resolving it. Measurements are marked as refined fractionation (▼), which narrows a fraction onto a question‐relevant subpopulation, or orthogonal measurement (○), which characterizes the shape of the distribution. Listed variations are illustrative rather than exhaustive. (B) Illustration of how a resolving measurement from (A), here a mineralization assay, resolves more when performed on a fraction than on bulk soil. On bulk SOM, the assay returns one broad distribution summarized by a single central tendency. Physical fractionation separates this into POM, free MAOM, and occluded MAOM, each with a narrower distribution (bulk distribution shown dashed for comparison). Applied to occluded MAOM alone, the same assay resolves internal structure that the fraction's single peak (dashed) conceals.

### 4.1 A Refined Approach to Physical Fractionation

POM and MAOM provide a conceptual structure from which new and refined fractionations can extend, targeting subpopulations whose composition, persistence, or behavior is more uniform than that of the parent fraction. In practice, this means extending the standard one‐step size or density fractionation with additional separation steps that isolate these subpopulations. Introducing this complexity through refined fractionation is not new methodological territory. The field has a long tradition of such work (e.g., Poeplau et al. 2018). The difference now is that broad acceptance of the dichotomous framework means POM and MAOM can effectively act as parent pools, providing a shared conceptual reference even where operational definitions differ (Leuthold et al. 2024). Those parent pools allow us to focus refinement along the axes of interest. Free and occluded POM, isolated by altering dispersion and the timing of density flotation, distinguish material along gradients of physical protection and persistence (Golchin et al. 1994; Six et al. 2000; Haddix et al. 2020). MAOM density classes, isolated by sequential density fractionation at increasing thresholds, have been hypothesized to distinguish material along gradients of mineral‐association strength and persistence (Sollins et al. 2009). Refinement is not limited to within‐fraction subdivision: combining size and density at finer resolution can more accurately separate POM and MAOM themselves, as in isolating the CHAOM (Figure 2; Samson et al. 2020; Leuthold et al. 2024) that straddles the conventional sand‐sized boundary. These examples show that refined fractionation is most useful when each added separation is tied to a specific axis of inference (Figure 3).

Lavallee et al. (2020) purposefully avoided prescribing a methodology, understanding that doing so would constrain the field's ability to adapt fractionation to the mechanistic questions and resources available to different researchers. Yet, despite the depth of current fractionation methods, much of the within‐fraction variability cannot be parsed using the existing techniques. For some subfractions, adapted or new methods are needed to resolve the relevant variation directly. For example, current hypotheses suggest free and occluded MAOM differ in composition, stability, and behavior (Fulton‐Smith et al. 2024). Methods that target this separation, through altered dispersion and wet‐sieving sequences or adapted aggregate fractionation, would let these hypotheses be tested directly. Even with these kinds of extensions, however, we also need to ask whether the existing set of fractionation approaches captures the axes of within‐fraction variability that we aim to resolve. For example, stable and exchangeable MAOM are well established conceptually (Figure 2; Jilling et al. 2018; Villarino et al. 2023), but current methods cannot isolate them effectively. Recent work suggests that our paradigmatic methods may even mobilize and further obscure these forms of MAOM rather than help to resolve them (Jilling et al. 2025). This limitation reflects the path dependency of physical fractionation: most current approaches rely on particle size, density, or dispersion behavior, even when the target property is exchangeability, mineral association type, accessibility, or persistence. Identifying additional physicochemical properties whose variation maps more cleanly onto within‐fraction axes may unlock substantial new resolution. Magnetic susceptibility, for example, separates organic matter associated with iron‐oxide minerals from that bound to silicate clays (Chiti et al. 2019), characterizing a within‐MAOM distribution defined by mineralogy that standard size‐density fractionation cannot capture. As we develop new methodologies aimed at resolving second‐order heterogeneity, the productive frontier requires methods novel enough to ask new questions, accessible enough for the field to use, and rigorous enough that their results can transfer across studies.

For such refinements to advance understanding rather than fragment the field, their trade‐offs must be explicit. Each step of methodological elaboration carries real costs in recovery, in metadata burden, in comparability across studies, and in the time and labor required to carry it out. These costs compound as the fractionation library available to researchers expands. As additional fractionation methods are adopted, shared methodological standards become imperative to preserve the shared understanding that the framework enabled and guard the science against misinterpretation and spurious findings. Chief among these are standards for calculating and reporting organic C and total N recovery. Each additional fractionation step risks solubilizing material that would otherwise be retained in a fraction, washing it into the aqueous phase instead. These losses accumulate as subfractions proliferate, compounding a baseline that is already variable, and often unreported (Plaza et al. 2019). Consistent reporting of recovery against independently measured bulk values is the minimum standard required for credible inter‐study comparison. How we quantify recovery (e.g., through laboratory replication), and the variability that fractionation itself introduces, are important methodological considerations for the field. Fractionation is rarely replicated in the laboratory. Replicating the procedure, rather than the measurement of the fractions, would help reveal how reproducible the separation is and how its variability might propagate downstream. Robust fractionation studies require more than recovery, however. Fractionation methods and soil properties interact, shaping the boundary between POM and MAOM in ways that depend on the soil being fractionated (Leuthold et al. 2024; Motsi et al. 2026). Sand content, in particular, appears to moderate inter‐fraction C transfer and contamination (Poeplau et al. 2025). The choice of heavy liquid can itself skew fraction C and N measurements (Plaza et al. 2019). As such, reporting basic soil properties, such as texture, bulk density, pH, and carbonate content, when available, is required for results to be interpretable and interoperable across studies (Billings et al. 2021). Robust metadata also enables direct comparison of conceptually similar fractions with different operational definitions, via transfer functions (Kpemoua et al. 2026). Together, these standards allow the methodological library to expand without disregarding the framework that gives it shared meaning.

The goal of adapting new fractionation methods should be to better resolve the second‐order heterogeneity within POM and MAOM. Subfractions are therefore best understood as extensions of these parent fractions that characterize the within‐fraction distribution, not as replacements for them. Defining them in relation to their parent fractions and reporting the operational basis of their separation preserves the semantic clarity the original framework provided while allowing for increased depth. Greater methodological resolution brings a greater burden of explanation. The more elaborate the fractionation scheme, the less accessible it is to the broader research community. SOM biogeochemistry, then, needs a balance: while some questions justify elaborate fractionation, the original two‐fraction framework remains a powerful means of inquiry for many questions. For example, in regions where SOM biogeochemistry remains understudied, such as the Global South and the tropics, characterizing POM and MAOM through a single‐step fractionation is itself a meaningful advance, extending the framework's reach to systems that global syntheses have largely missed (Sun et al. 2026). Just as experimental design is chosen to give an inquiry the statistical power needed to resolve a specific question, fractionation methods should be chosen for the inferential power they provide for the question at hand.

### 4.2 Characterizing Fractions Through Orthogonal Measurements

Some forms of within‐fraction heterogeneity simply cannot be parsed by separating soil into finer subpopulations. Variation in composition can exist within a single clay particle, at scales below any method of physical fractionation (Wan et al. 2007; Vogel et al. 2014), and the hypothesized zonal arrangement of organic matter on mineral surfaces implies that persistence can vary at the same scale (Kleber et al. 2007). Process dynamics like turnover and decomposition rates are likewise invisible to methods that capture only state at a single point in time. Together, these limits define an information ceiling for physical fractionation: each resulting fraction, regardless of how finely split, is a composite fraction taken at the moment of sampling. In these cases, characterizing the distribution along the relevant axis becomes more useful than further subfractionation, placing each subpopulation within the broader distribution rather than treating it in isolation. Pairing fractionation with orthogonal information offers a way past this limit, whether through within‐fraction characterization, environmental context, or process‐based modeling (Figure 3).

Within a fraction there is some heterogeneity that is better characterized directly than further partitioned. Mid‐infrared spectroscopy resolves molecular composition across the carbohydrate, aromatic, and aliphatic regions, capturing degradation state and source attribution that bulk C and N measurements cannot (Calderón et al. 2011; Schiedung et al. 2025). Pyrolysis‐GC/MS extends this with finer molecular detail, and hydrogen pyrolysis (HyPy) can isolate PyOM within bulk soil and its fractions (Lavallee et al. 2019). Together, these methods and others like them let researchers look back at how a fraction formed and forward at how its composition shapes behavior. Persistence within a fraction spans orders of magnitude, and the balance between fast‐ and slow‐turnover material shapes how the fraction will respond to disturbance, warming, or land‐use change. Thermal methods such as ramped pyrolysis (Sanderman and Grandy 2020; Stoner et al. 2023) and Rock‐Eval (Barré et al. 2024) characterize this distribution directly. Sorption isotherms (Abramoff et al. 2021), priming assays (Jilling et al. 2021), and destabilization experiments (Li et al. 2021) are examples of methods that characterize the within‐fraction distribution of reactivity. By separating readily mobilized material from material that stays bound under the same conditions, we resolve which subpopulation within the fraction is biologically and chemically active. Mineralization rates of isolated fractions (Sollins et al. 1984; Leuthold, Lavallee, et al. 2025) measure respiration and nutrient release for each subpopulation, clarifying functional contributions that fraction identity cannot capture. Within‐fraction characterization extends the POM‐MAOM framework's domain of validity so that fractions can be better linked to functions.

Fractions describe the state of SOM at sampling, but its trajectory depends on environmental context the separation does not encode. Within the MAOM fraction, for example, residence times can differ by up to an order of magnitude across mineralogical gradients, driven by variability in iron oxide content and reactive mineral surface area (Heckman et al. 2018; Wattel‐Koekkoek et al. 2003). POM decomposition rates have been shown to vary across temperature and moisture gradients (Haddix et al. 2020; Lugato et al. 2021). Physical fractionation by itself may reveal these differences in central tendency, but it is poorly suited to explain them. Closing this gap requires integrating fraction identity with the environmental context that governs its trajectory (Angst et al. 2023). Frameworks that integrate soil mineralogical setting (King et al. 2023) or MAOM saturation status (Georgiou et al. 2025) provide examples of contextual integration and should be built upon to include other drivers, such as climate and vegetation impacts. Predictive inference about fractions thus requires fraction identity, within‐fraction structure, and environmental context together.

The same logic underlies process‐based models. Well‐reasoned models can address mechanistic questions that measurement alone cannot, provided that they are built from first principles theory that represents the conceptual pools as faithfully as current understanding allows. Models that encode mechanism support counterfactual reasoning about how fractions would respond under conditions outside the observational record, such as whether a given pool will persist or destabilize under sustained warming (Parton et al. 1988; Abramoff et al. 2018; Zhang et al. 2021). A distributional view of SOM can sharpen these inferences. Within‐fraction distributions can be used to parameterize the rate constants governing modeled reactions more accurately than a central tendency, because they carry the full range of a fraction's behavior, not only its average. Environmental context and subpopulation characterization can better constrain the parameters that govern SOM formation, persistence, and turnover. The reliability of these counterfactuals, in turn, depends on grounding the next iteration of process‐based models in measured fraction data and mechanistic theory, and testing them against independent experiments (Baveye 2025). Here, what the framework supplies to these models expands from each fraction's central tendency to its full distribution, offering a shared vocabulary for testing hypotheses about variation within fractions as opposed to only about differences between them. Within‐fraction characterization, environmental context, and process‐based modeling each build on and extend what fraction identity supports.

## 5 Conclusion

Physical fractionation has supported decades of research into SOM heterogeneity, and the POM‐MAOM framework codified by Lavallee et al. (2020) gave that work a shared foundation. This distinction between particulate and mineral‐associated organic matter maps onto our contemporary understanding of how C and N enter, persist in, and cycle through soils. When we isolate these fractions, we approximate the conceptual pools via operational cutoffs, which produce internally heterogeneous composites whose properties are better understood as distributions than as fixed, singular values. Advancing the next generation of questions means acknowledging this distributional structure, characterizing it, and narrowing it along the axes that specific inquiries demand (Figure 1). Here we emphasize two complementary approaches to resolving these distributions: (1) refining fractionation along these axes (such as separating free from aggregate‐occluded POM and MAOM, distinguishing stable from exchangeable MAOM, or isolating CHAOM and PyOM components; Figure 2), and (2) pairing fractions with orthogonal measurements. Together, these refinements resolve the within‐fraction distribution that bulk measurements integrate over, deepening understanding of SOM dynamics and linking conceptual pools to ecosystem function (Figure 3). The framework's continued value depends less on how finely we can divide soil than on why we divide it, allowing the question to determine which axis we aim to resolve. We encourage the community to take up this inquiry‐driven approach, letting the questions, and the resources at hand, determine when a fraction is best left whole and when it must be resolved further. Tailoring our fractionations to the specific inference we aim to draw will allow us to link fraction to function and inform the future stewardship of soil C (Janzen 2024). A separation made for its own sake yields more data but does not necessarily yield more understanding.

## Author Contributions

**Sam J. Leuthold:** conceptualization, writing – original draft, visualization. **Jocelyn M. Lavallee:** conceptualization, writing – review and editing, visualization. **M. Francesca Cotrufo:** conceptualization, funding acquisition, writing – review and editing, supervision.

## Funding

This work was supported by the USDA (Grant 2025‐68012‐44236). Jocelyn M. Lavallee's contribution to this work was supported by Environmental Defense Fund with an award from King Philanthropies.

## Conflicts of Interest

M. Francesca Cotrufo is a co‐owner of Cquester Analytics LLC, a service analytical facility that provides analyses of SOM, POM, and MAOM. Sam J. Leuthold has acted as a paid consultant to Cquester Analytics. Jocelyn M. Lavallee declares no conflicts of interest.

## Data Availability

The authors have nothing to report.
